# The characteristics of the surface structure obtained in a low plasticity burnishing process in which the jumping wave occurred

**DOI:** 10.1038/s41598-025-02449-2

**Published:** 2025-05-21

**Authors:** Stefan Dzionk, Bogdan Scibiorski

**Affiliations:** Institute of Manufacturing and Materials Technology, Faculty of Mechanical Engineering and Ship Technology, Gdansk University of Technology, Gdansk, 80-233 Poland

**Keywords:** Jumping wave, Surface layer, Low plasticity burnishing process, Engineering, Materials science

## Abstract

Jumping wave formation in front of the processing tools occurs during low plasticity burnishing methods. It significantly disturbs the plastic deformation of surface structures process. The wave causes also additional structures to appear on the processed surface such as additional dales, seems and laps. Furthermore, the rise of processing burnishing force causes significant increase the depth of the surface structure dales which are caused as a result of increase of the slippage rate of material layers in the jumping wave. The results are presented based on 3D profilograms and metallographic studies of the material structure in the surface layer.

## Introduction

Increasing demands on the manufacturing process in terms of energy consumption and waste generation mean that waste-free technologies are increasingly being used^1^. One of the waste-free finishing technologies is the burnishing method. This method consists of plastically deforming the surface of the component to be processed. There are many methods of burnishing, differentiated by: sliding or rolling pressing; by shape of pressing elements, i.e. rollers, balls, barrels, etc.; by way of tool pressing, i.e. rigid contact pressure (with constant position of burnishing elements) or elastic contact pressure (constant value of tool pressing force); smoothing and strengthening. The burnishing process is applied to various materials in both soft (≤ 40 HRC Rockwell grade) and hardened states (> 40 HRC). When burnishing soft materials (≤ 40 HRC) there is usually a wave of the workpiece material in front of the tool, which moves with the tool. In the resulting wave of the material, plastic deformation occur, including slippage of the material between successive layers which cause creation additional geometric structures in the surface layer of the component to be burnished. Deformation models presented in the literature^1,2^ usually do not take into account the wave effect and the changes in surface structure that are caused by it. The size and type of these deformations depend on the properties of the workpiece material, and the machining parameters and their influence on the structure of the surface after burnishing. The process of wave formation and its effect on machining results has been previously process of wave formation and its effect on machining results has been previously presented by the authors^2^. This article is continued research and presents new aspects of the phenomena occurring in the jumping wave during the burnishing process.

### Literature review

The current state of knowledge on burnishing technology trends and research methods has been previously discussed^2^. Contemporary literature does not directly address the impact of the burnishing process on the phenomena occurring in the wave before the tool. Both in experimental research^3–7^ and numerical modelling^8–12^, the material of burnished elements is typically treated as a homogeneous structure, and the influence of deformation-related phenomena on surface texture is often overlooked. Surface characterization is primarily based on the Ra (Sa) parameter, which provides only an average measure of roughness. However, specific features, such as deep valleys, may remain undetected using this parameter.

Nevertheless, some studies contain surface structures that may result from phenomena occurring in the wave before the tool. These structures, although present in published results, are often interpreted in the context of other factors and remain insufficiently discussed. The following review highlights selected studies that include observations suggesting the impact of the wave in the burnishing process, even if these aspects were not the main focus of those works.

V. Schulze et al.^13^ present a comprehensive review of surface layer properties resulting from hardening and strengthening processes such as burnishing and shot peening. While the presence of a wave before the tool is acknowledged, its impact on surface characteristics is not explicitly linked. The study includes valuable images of burnished surfaces, where signs of material slippage within the wave can be observed as surface imperfections. These are interpreted as effects of excessive burnishing force.

In the review article^14^ on burnishing thick coatings, various surface phenomena are analysed. However, the role of wave formation before the tool and its specific influence on the final surface condition is not discussed in detail.

V. P. Kuznetsov et al.^15^ investigate nanostructuring burnishing as a subsurface shear instability phenomenon. Their surface images reveal defects associated with sliding burnishing, where increased load intensifies discontinuities in the subsurface layer. These defects are attributed to quasi-viscous flows in the plastic deformation zone, dependent on loading conditions and tool friction.

T. Dyl et al.^16^ present 3D profilographs of burnished coating alloy surfaces. Certain features in their surface topography suggest the influence of phenomena occurring in the wave before the tool. Additionally, some profiles contain cavities with structures characteristic of material layer slippage.

S. Świrad and P. Pawlus^17^ examine burnished surfaces using SEM imaging under 20 MPa pressure. Their images reveal wavy patterns formed due to overlapping and compression of displaced material layers, resembling effects observed during burnishing with wave formation.

A. M. Abrão et al.^18^ provide a set of surface images illustrating changes due to burnishing force. These images show typical structures resulting from material layer slippage during the process, with more pronounced pressing effects and serrated edges at higher loads. The study attributes these formations to excessive burnishing pressure, which may lead to surface flaking.

A. Sequera et al.^19^ analyse the surface condition of Inconel superalloy after ball burnishing. The many images illustrate the topography of surfaces subjected to different burnishing forces. Other features are commented on in these materials, nevertheless, features associated with material slippage may be noticed.

G. V. Duncheva et al.^20^ primarily characterize surface roughness using the Ra parameter. However, their surface images reveal deep valleys whose contour changes with successive tool passes, suggesting interaction with the wave phenomenon.

In their review, A. Raza and S. Kumar^21^ present SEM images of surface roughness, where displaced material layers from the wave during burnishing are discernible.

A. de Oliveira^22^ describes a surface flaw resembling an overlapping material layer, where the serrated edge suggests bending of the deformation wave. This flaw was observed on surfaces burnished at 40 MPa pressure and a speed of 100 mm/min.

K. Low and K. Wong^23^ examine polymer material surfaces, where features indicative of wave formation and material slippage during burnishing are present.

Surface defects resulting from the wave phenomenon created by the burnishing tool have also been documented in numerous other studies^24–28^. These works collectively indicate that while certain surface structures observed in experimental research may stem from wave-related phenomena, this aspect remains underexplored. A deeper investigation into these effects would enhance understanding of the burnishing process and its influence on surface integrity. Despite the presence of scattered references to these phenomena, there is a notable lack of comprehensive studies that thoroughly describe and explain the nature, causes, and consequences of surface defects induced by the wave effect. Existing research typically does not provide a detailed classification of the defects, nor does it attempt to correlate them with specific process parameters in a systematic manner. Furthermore, the available studies often do not investigate the microstructural changes within the affected regions, leaving gaps in understanding how these defects develop at different force levels, tool geometries, or material properties.

Recognizing this research gap, the present study aims to explain and describe the wave-induced surface defects, focusing on their formation mechanisms, evolution, and potential implications for the burnishing process. By analysing these phenomena in greater detail, this research seeks to establish a more profound understanding of the interaction between the tool and the material during burnishing. Such insights will not only contribute to improving the predictability of surface quality but also enable better process control and optimization. A deeper knowledge of these mechanisms may allow for the development of adaptive burnishing strategies that minimize undesirable surface effects, ultimately leading to improved functional properties of processed components.

### Burnishing process

**Fig. 1:**
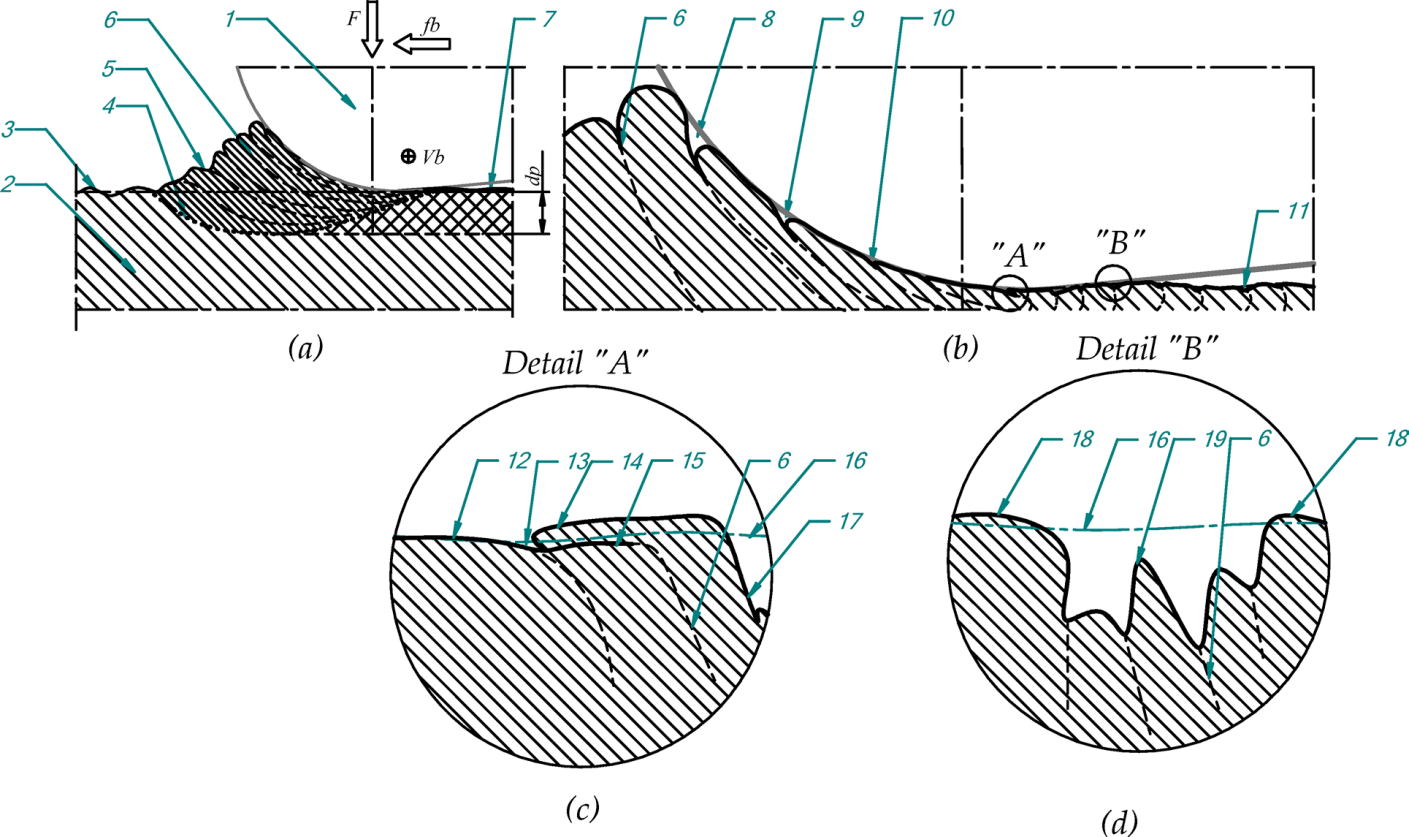
Scheme of burnishing process with the jumping wave effect; (**a**) general view; (**b**) detail phenomena occurs in the process; (**c**) fold as an overlapping material on the surface, (**d**) dale as a detail of surface structure; 1- burnishing tool, 2- workpiece, 3- surface before process, 4- plasticization outline of the material, 5- wave surface; 6- material sliding line, 7- surface after burnishing, 8 ÷ 10- consequence steps of creating flaws as a result of the material sliding, 11- flaw on the burnished surface as a result of material sliding, 12- burnished surface, 13- trace of a seam on the surface, 14- overlapping material, 15- adhesion join line of the overlapping material, 16- reference line of the surface, 17- surface of the dale, 18- raised surface in the dale environment, the irregularities dale inside; F- burnishing force, fb- feed of burnishing, Vb- velocity of burnishing, dp - the depth of changes in the material connected with wave formation.

Figure 1 shows a schematic representation of the burnishing process during which a wave is formed on the workpiece material surface of the processing part in front of the working tool. This process causes a number of changes on the surface of the workpiece. The pressure from the burnishing tool causes the material to plasticize. The plasticized material is moved in front of the tool. In this plasticization zone, tensions occur that continuously cause plastic deformation of the material located in tool environment with the significant deformations generated in the wave of the material where the main form of deformation is slippage of the material layers. It is difficult to find data on deformations as well as forms and directions of material slip during burnishing in the literature, but some information on the course of material deformation during cutting process e.g^29^. may be useful for analysis of burnishing process. The displacement of the layers causes faults formation on the surface. The resulting faults create an additional structure of irregularities that moves under the tool during the burning process.

The pressure exerted by the tool partially reduces the irregularities formed by the faults but it does not completely eliminate this. In this way on the surface are created additional peaks and dales which do not result from previous machining. The degree of reduction in recesses created by slipping layers of material mainly is depended on the pressure loads exerted by burnishing tool. In this process the tool shape is relevant because it determines the local pressure contact area from tool and workpiece. Depending on the value of burnishing forces and shape of the tool, the various forms and numbers of additional dales and overlapped material structures are created on the surface of the burnishing element. This process is schematically presented in Fig. 1b. Figure 1c,d show schematically typical additional structures formed on the surface due to the above-mentioned phenomena. These structures are formed by the displacement of layers of material in the wave before the tool as a result of shear stresses occurring there. As the material has a heterogeneous structure and there are grains with different properties, the sliding process of the layers is not regular. This causes the layers of material to align differently with each other, resulting in the formation of folds and deep cavities. Figure 1c schematic shows a fold. Such the fold is formed when a layer of material is pushed above the surface. Next, it is pressed against the surface by the burnishing element.

Pressing down causes the folds to be flattened on the surface. After flattening a part of the material is only connected with the surface by adhesion force as other bonds in the material have been broken. In Fig. 1c, the line (item 15) presents schematically the adhesive bond of the fold with the material. The high strain associated with pressing the fold material by the tool into the surface causes great deformation of it and edges of the fold structure become serrated and slightly raised. The more increase pressures may cause the fold fragments of material to detach from the surface creating a flaking phenomenon. Detail B in Fig. 1d shows a schematic representation of the resulting cavity on the surface. The displacement of material in the wave also results in the formation of faults creating the cavities. These structures create in the wave, deform under the pressure of the burnishing tool, which significantly reduces their shape and size. The particular feature of such depressions are the visible irregularities in the bottom and slightly raised material on the edge environment. Bottom irregularities caused by material layer slippage and can be in some places very deep. These features schematically are presented in Fig. 1d marked by the items 18 and 19. In the literature^30^ are described research works that present decrease in the depth of cavities with an increase in burnishing force. The measurements taken in such tests are usually contact measurements, and should be taken into account because these methods of measurement filter out small and deep cavities.

## Materials and methods

**Fig. 2:**
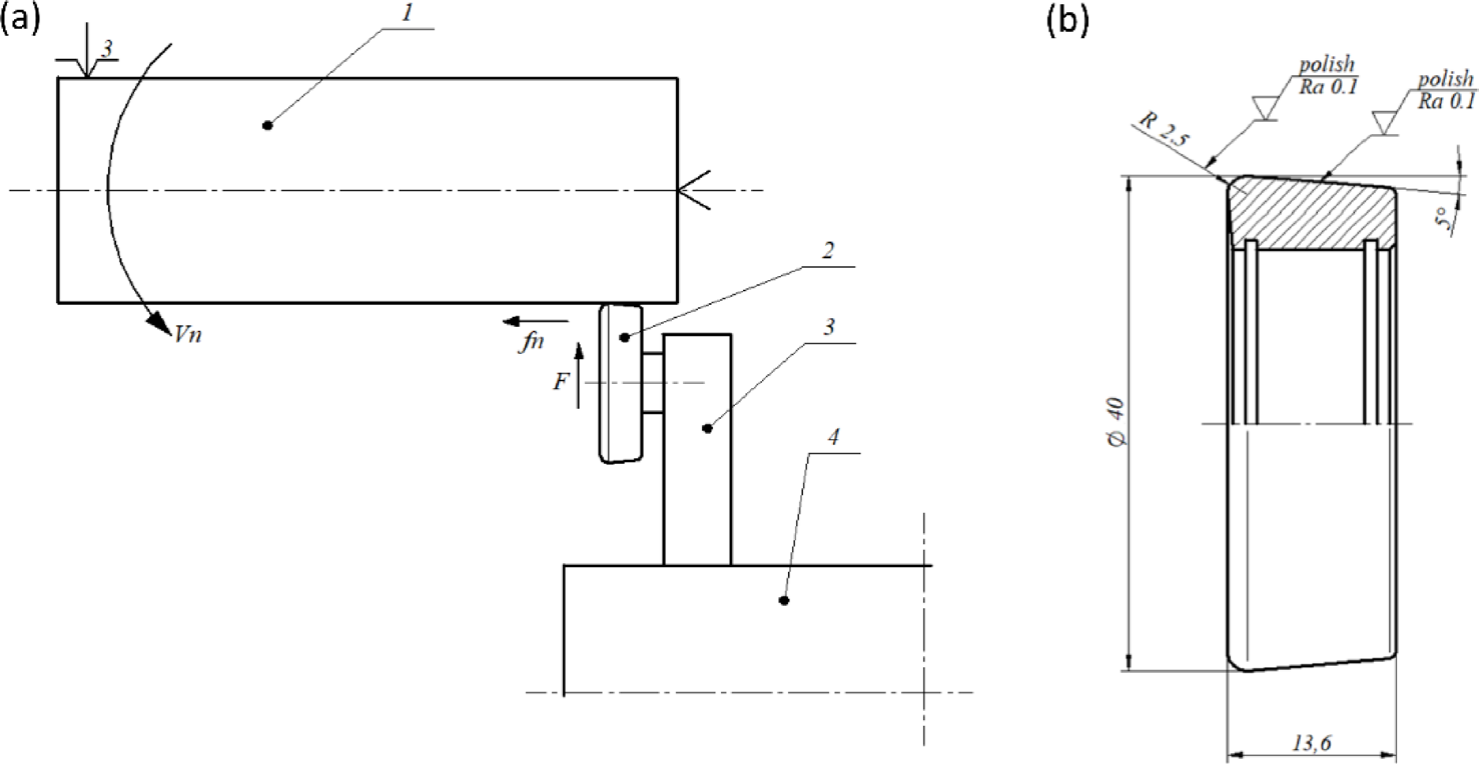
Schematic of the burnishing process (**a**) and sketch of the burnishing roll (**b**): 1 – machined shaft, 2 – burnishing roll, 3 – handle of the burnishing tool, 4 – tool post, Vn – burnishing speed, fn – burnishing feed, F – burnishing force.

**Fig. 3:**
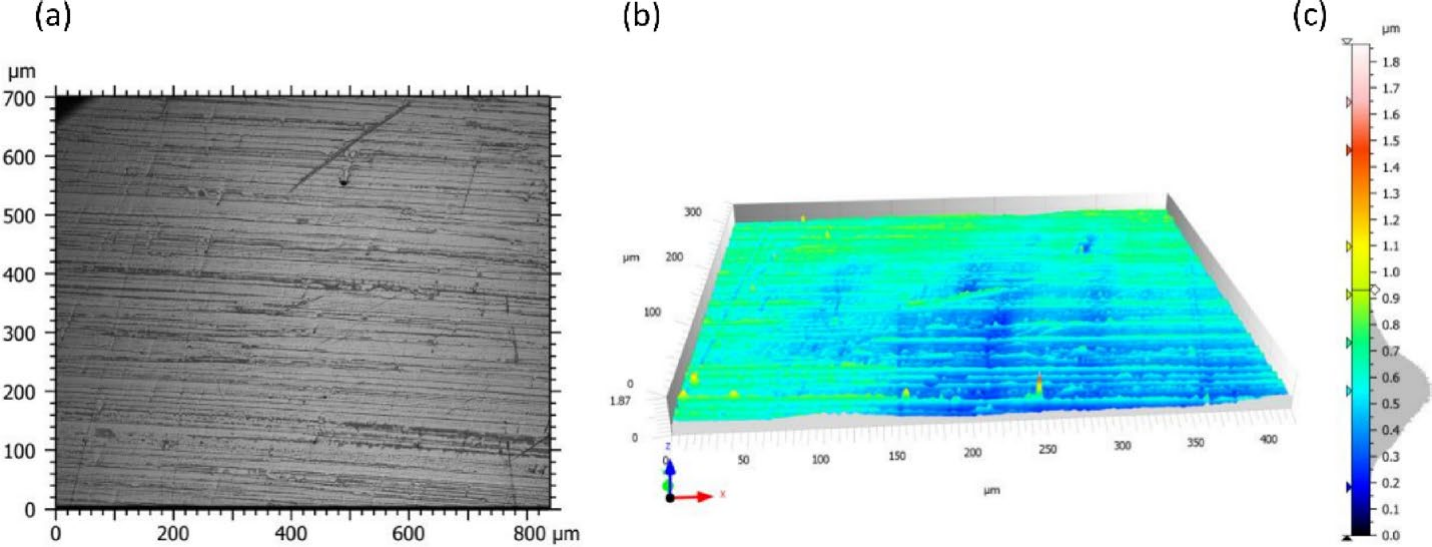
Surface structure of the burnishing roller: (**a**) photograph of the surface, (**b**) profilogram. (**c**) scale and height distribution curve.

The tests were conducted on turned samples in the form of a shaft with a diameter of ø 97 mm and a length of 350 mm. Before the burnishing process, the samples were pre-prepared by turning at a cutting speed of Vc = 61 m/min and a turning feed of ft = 0.3 mm/rev. Samples from the same batch were used in a previous study^2^, and the geometric surface structure, along with photographs of the metallographic structures. The samples were made of steel 1.0562, and its chemical composition was confirmed using spectral analysis. The results of the chemical analysis and the mechanical properties of the steel are presented in Table 1. During the tests, the samples were clamped in a three-jaw self-centering chuck with tailstock support, which ensured their stability during the process. The burnishing process was carried out on a “ZMM Sliven 400” universal chuck-tailstock lathe, and the sample mounting scheme is shown in Fig. 2a. Prior to the tests, the diameter deviations along the entire length after turning were checked and did not exceed 0.01 mm. The burnishing tool was fixed in the lathe tool post and consisted of a roller made of bearing steel 100Cr6 (1.3505) with a hardness of 63 HRC ± 1 HRC. The geometry of the burnishing tool was characterized by the following parameters: a roller diameter (Dbt) of 40 mm, a toroidal radius (Rbt) of 2.5 mm, and half of the apex angle of the cone (γ) of 5°. The shape and dimensions of the roller, as well as the fixing method of the burnishing tool, are presented in Fig. 2a,b. The roughness of the burnishing roller is presented in Fig. 3; Table 2. Analysing the surface structure of the burnishing roller, it can be observed that its features are characteristic of polishing processes. The surface exhibits scratches, which are traces left by abrasive grains from the polishing paste. The analysis of the roller’s surface roughness profile shows a symmetric distribution in the amplitude density curve, as illustrated in Fig. 3c. In addition, the surface contains individual elevations that may result from residual abrasive particles embedded in the surface, a phenomenon typical for polished surfaces. In the case of the burnishing roller, such residual abrasive grains after the polishing process are difficult to detect and are barely visible in Fig. 2a,b. All measurements were carried out in accordance with machining process guidelines, and the applied process parameters are summarized in Table 3.

All measurements were conducted following machining process recommendations, and the applied processing parameters are summarized in Table 4.

As a result of the application of three levels of forces, the sample diameters changed slightly, which is presented in Table 3. During the process the machine oil type L-AN 46 acc. (ISO 3448) was used.

**Table 1:** Material data.

EN	C [wt%]	Mn [wt%]	Si [wt%]	*P* [wt%]	S [wt%]	Cr [wt%]	Ni [wt%]	Al [wt%]	Cu [wt%]	Nb [wt%]
1.0562	0.14	1.36	0.17	0.018	0.009	0.076	0.039	0.033	0.095	0.023
	Parameter	Hardness (in a soft state)		Tensile strength (Rm)				The yield strength (Re)		
	Unit	HB (Brinell scale)	HRC (Rockwell scale)	MPa				MPa		
	Value	220	< 20	490–630				335		

**Table 2:** The 3D parameters of the surface burnishing roller, acc. ISO 25,178^31^.

Height parameters	Value	Unit	Description
Sa	0.10	µm	Arithmetic mean height
Sq	0.12	µm	Root-mean-square height
Ssk	0.06		Skewness
Sku	2.99		Kurtosis
Sp	1.32	µm	Maximum peak height
Sv	0.54	µm	Maximum dale height
Sz	1.87	µm	Maximum height

Metallographic samples were made in the same conditions as in the described article. The observations were continued on an OLYMPUS BX51 microscope with OLYMPUS Stream Motion software. The technical details of the study are described in the article^2^ Micro-hardness measurements were carried out on a FM-800 device FUTURE-TECH. CORP.

## Results and discussion

**Fig. 4:**
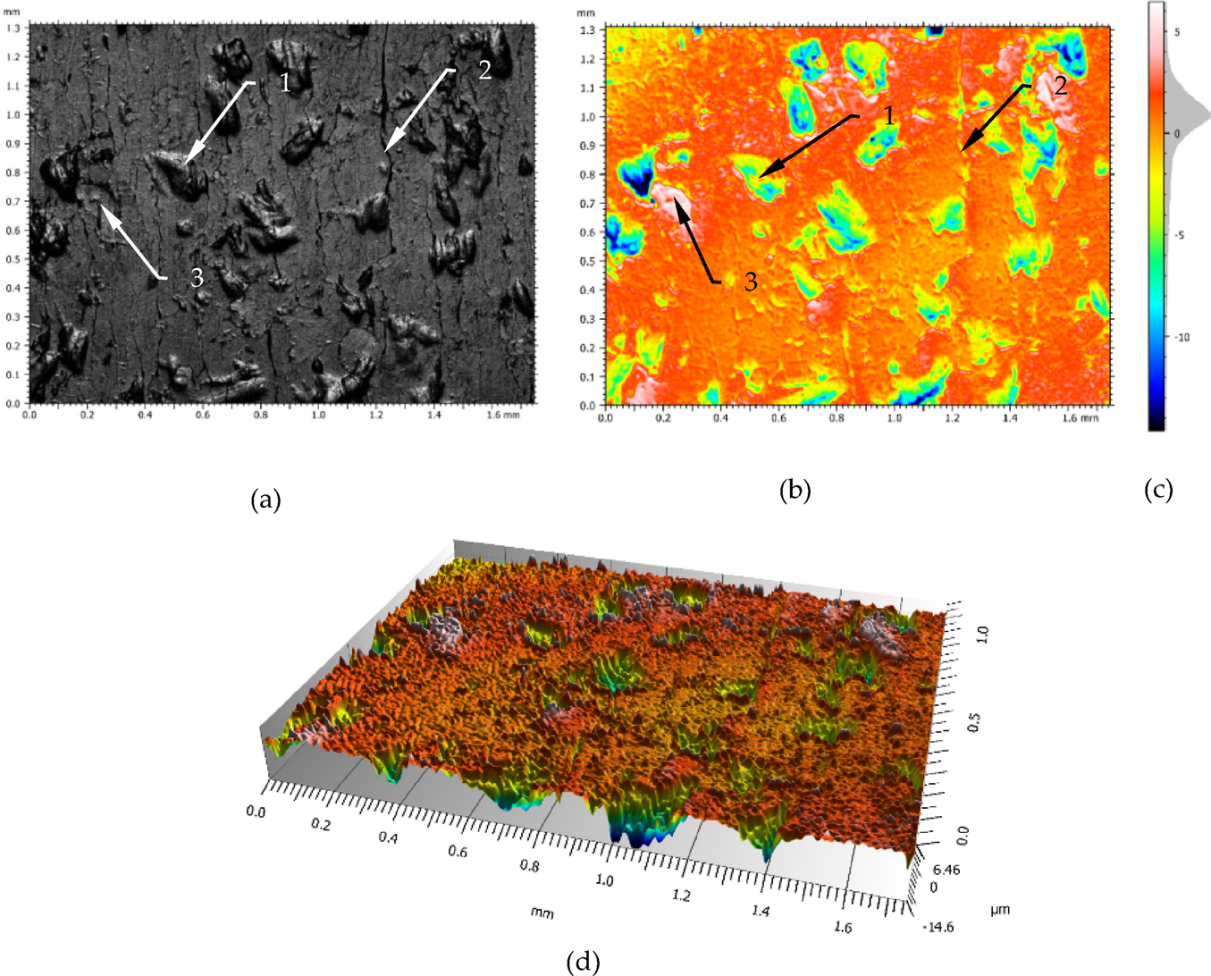
The surface structure after burnishing with a force of 550 N: (**a**) photograph of the surface, (**b**) profilograph, (**c**) scale and height distribution curve, (**d**) 3D profilograph; 1- deep dale of the surface, 2- line of seams, 3- fold on the surface.

Figure 4 presents the surface texture after burnishing with the parameters defined in Table 3, line 1 (Force 550 N). The burnishing force was not very high whereas on the surface can be noticed the effects of phenomena occurring in the wave in front of the tool. There are: large number of depressions (marked 1), lot of seams traces (marked 2) and places with the laps (marked 3).

These structures are not result from the preparation of the material for burnishing (the detail research results of the surface after turning machining are in^2^), but are caused by the way of deformation and slippages of the material in the wave in front of the burnishing tool. The shape and position of the amplitude density curve which is shown in Fig. 4c indicate that some cavities are relatively deep. In Fig. 4d may be seen visible additional irregularities inside of the dale. The surface roughness parameters values characterized this area are presented in Table 4. The value of the Sa parameter is relatively large as for finishing. This is due to too low value of the burnishing force. This value of force (550 N) caused that the surface was not sufficiently deformed plastically, which resulted to remain them more cavities on the processed surface and slightly irregularities in the plateau areas. The parameter Ssk indicates that the ordinate distribution is shifted towards the peaks side. Another characteristic element visible on the surface are the creases (item 2 in Fig. 4). A detailed observation of Fig. 4a shows that there are a lot of surface marks of creases. Due to the irregularity of their occurrence, they may not be residues, e.g. from structures occurring during turning. It is more likely that they result from the pressing of faults that are created in the wave by the slippage of the material. In Fig. 4a it can be seen that slippage of the material layers in the wave occurs very frequently. A significant slippage of material layers in the wave causes creating a fault which next is cold rolled. In the result of this phenomena are visible seams on the surface (2), while slipping to a lesser range creates small seams which are distributed over the surface quite densely. This type of defect is difficult to see in Fig. 4.

**Table 3:** Processing parameters.

No of sample	Diameter after turning (before burnishing) [mm]	Diameter (after burnishing) [mm]	Force F[*N*]	Burnishing speed Vn [m/min]	Feed burnishing fn [mm/rev]
1	97.00 ± 0,02	96.87 ± 0,02	550	30,5	0,2
2	96.80 ± 0,02	96.70 ± 0,02	800	30,5	0,2
3	96.08 ± 0,02	95.85 ± 0,02	1150	30,5	0,2

**Fig. 5:**
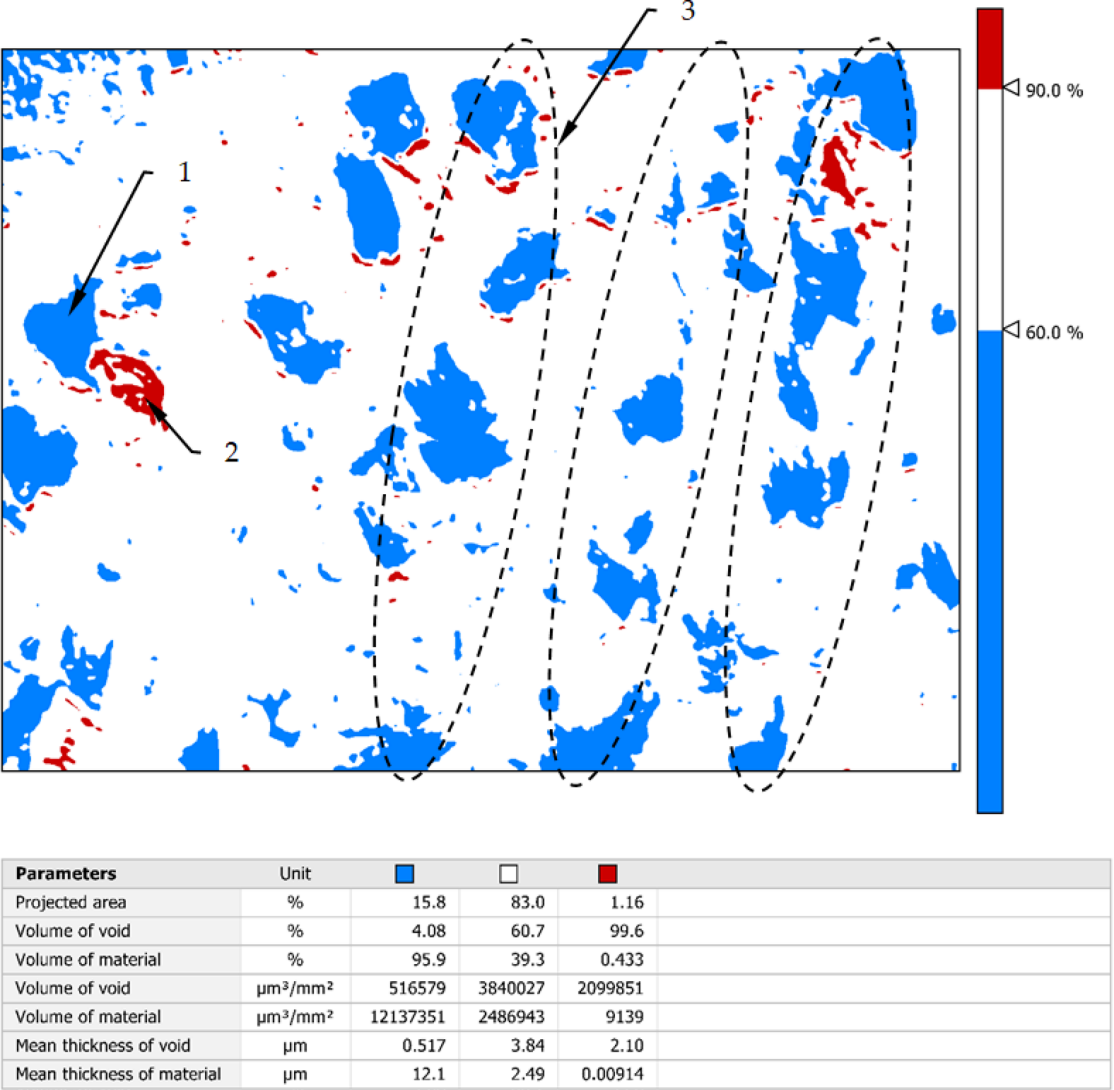
The peaks and dales structure the surface burnished with a force 550 N: 1- deep dale, 2- height peak, 3- areas of the layers slipping.

Figure 5 shows an analysis of the occurrence of significant dale and peaks in the area defined in Fig. 4. The height range of the irregularities occurring in this area has been divided into three levels: a zone of dales, a zone of peaks and a central zone.

The thickness and position of the central zone relative to the height range of the roughness is corresponded with the location of the amplitude density curve which is presented in the Fig. 4c. The central zone in Fig. 5 occupies more than 80% of the area. It is designated as the white zone and has a height equal to 30% of the Sz value which recalculated is 6.33 μm. From comparing parameters for the central zone: Mean thickness of void 3.84 μm to mean thickness of material 2.49 μm can be concluded that the profile in this zone is not symmetrical, and the dominant element are dale. The dales zone in this Figure covers an area of 15.8% and it is relatively large area. The height of this zone shall be 12.66 μm (60% Sz parameter). By contrast, by comparing it to the parameters: mean thickness of material (12.1 μm) it can be concluded that the average depth of these depressions is small and this is also indicated by the parameter mean thickness of void (0.517 μm). The high range of this zone and the small average values mean that cavities of considerable depth are rare in this area. The peaks area of 1.16% has a height of 2.11 μm. In the Fig. 5 may be seen that peaks occur mainly in the whereabouts of the dales. Such a value of this parameter indicates that these irregularities will wear out very quickly during operation and usually they are not taken into account in floor shop practice.

The element marked 2 in Fig. 5 is probably a material fault that has pressed on the surface which could arise from too large material deformation by the slipping and then was pressed into the surface during the burnishing. Furthermore, may be seen that the cavities’ structure has a dominant direction of inequality. This structure may be connected with the material deformation direction as a result of the moving wave in front of the tool. This direction is resulted mainly from the burnishing parameters such as velocity and feed.

**Table 4:** The 3D parameters of the surface burnished with a force 550 N, acc. ISO 25,178^31^.

Height parameters	Value	Unit	Descpription
Sa	1.89	µm	Arithmetic mean height
Sq	2.76	µm	Root-mean-square height
Ssk	−1.86		Skewness
Sku	7.47		Kurtosis
Sp	6.46	µm	Maximum peak height
Sv	14.6	µm	Maximum dale height
Sz	21.1	µm	Maximum height

**Fig. 6:**
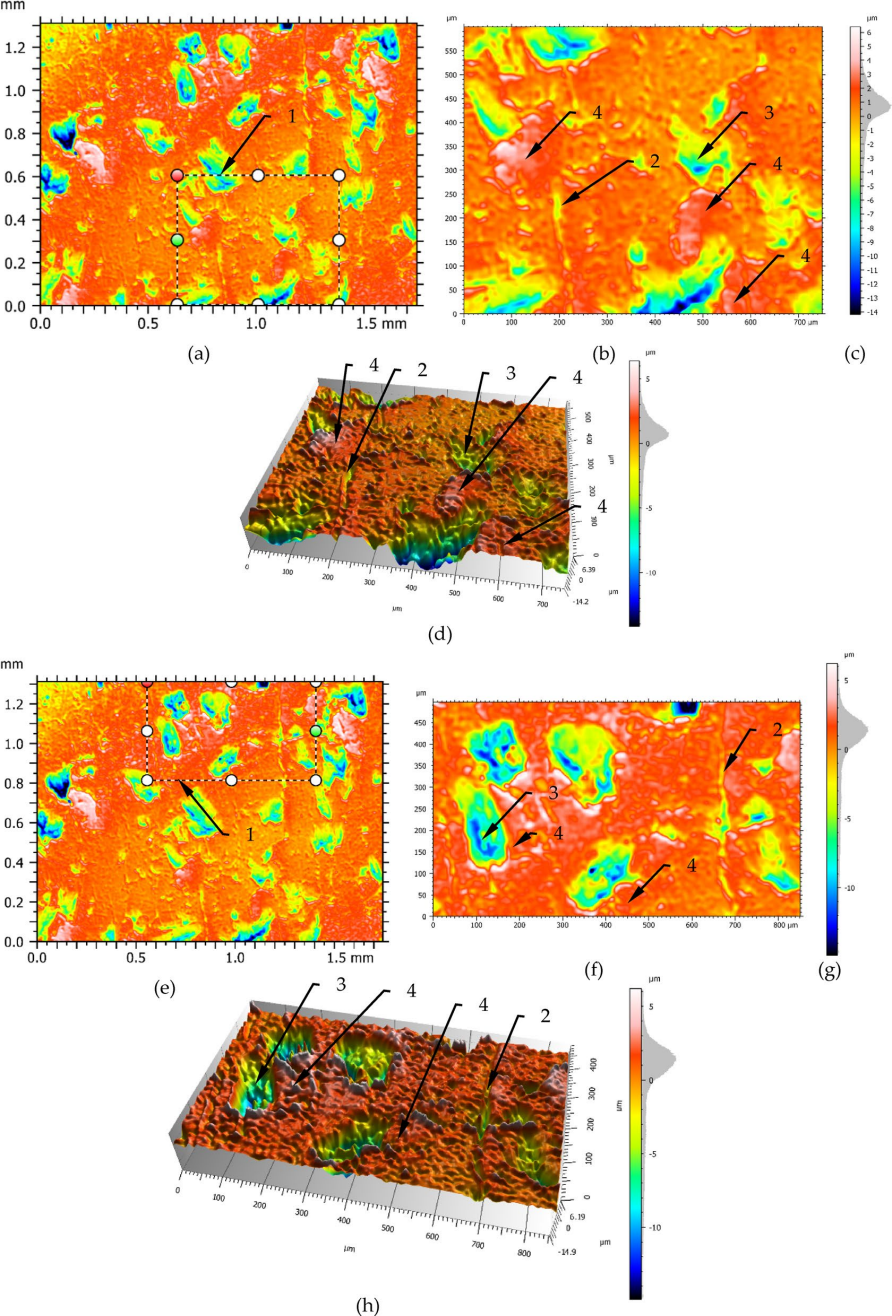
Surface structures after burnishing with a force of 550 N: (**a**), (**e**) the site of excision of the structure, (**b**), (**f**) enlargement of resected structures, (**d**), (**h**) 3D view of cut-out structures, (**c**), (**g**) scale and height distribution curve; 1- excision boundary, 2- line of laps, 3- deep dale, 4- pressed-in folds.

For a more precise surface characterization, magnifications of selected surface structures are shown in Fig. 6. This Figure presents an analysis of selected areas of the surface structure are shown in Figs. 4 and 5. In the magnified Fig. 6 can be seen easer the defects on the surface, in the form of laps (4) and seams (2).

**Fig. 7:**
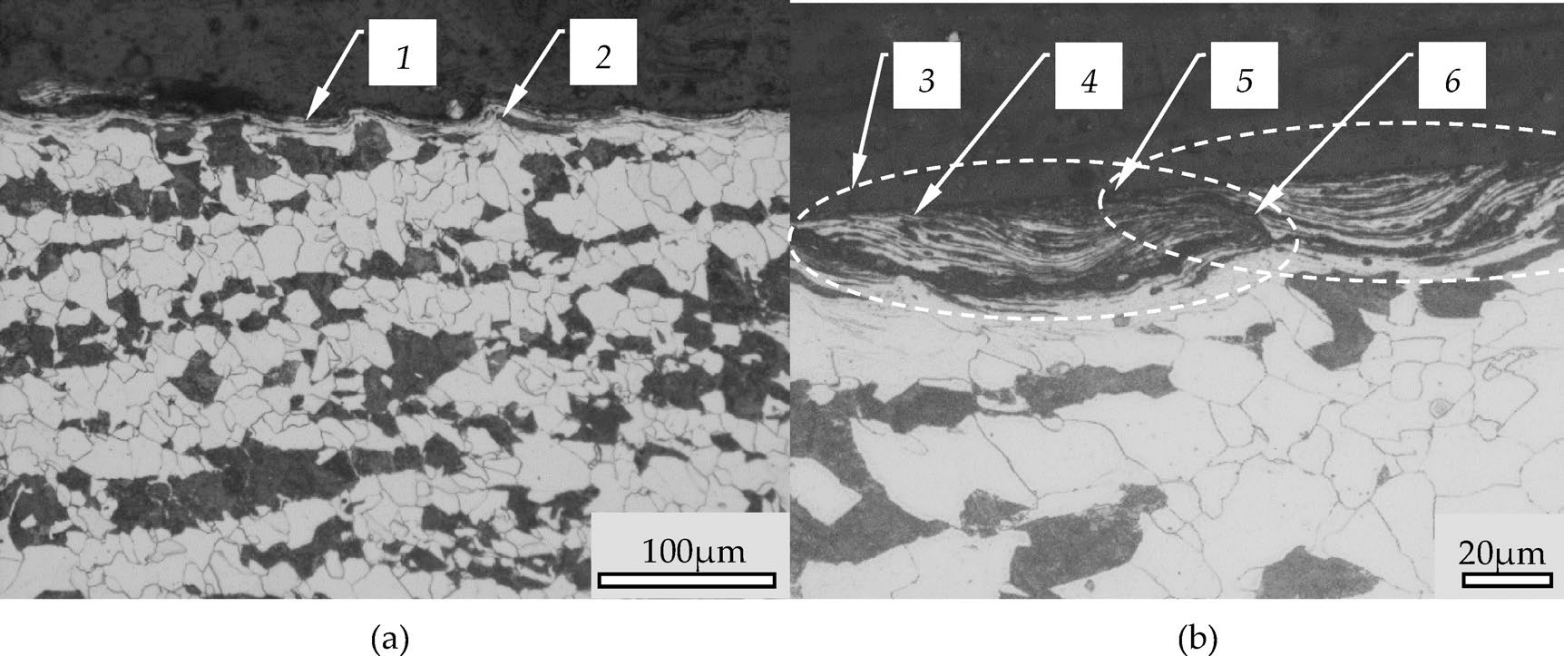
The cross-section of the surface after burnishing (burnishing feed *fn* = 0,2 mm/rev, radius of burnishing tool *Rbt* = 2,5 mm, *fn*/*Rbt* = 0,08): 1- burnished surface, 2- translated material as a slipping in the wave, 3- fold of the slipped material, 4- fold surface, 5- folds structure, 6- seams between folds.

Figure 6 shows enlargements of selected segments of the testing area. The enlarged area presented in Fig. 6b,f is marked with a dashed line (1) in Fig. 6a,e. On the analysed figures may be seen that on the central surface are irregularities in the form of seam lines (item 2). There are areas with overlapping material (item 4). Moreover, there are plateau areas which also show some surface structure. The bottom of the cavities (e.g. item 3 Fig. 6f) is differentiated in terms of the irregularities that occur there. Small areas with considerable depths of up to 15 μm may be seen there. From analysing the amplitude density diagrams can be seen that most of the profile ordinates occur in the − 1 μm to 3 μm range. On the other hand, very deep depressions exceeding 10 μm occur very rarely and may occur micro-cracks on the boundary of layer slip. In the Fig. 6 can be seen additional structures created as a result of pressing an additional material into the surface (item 4). There are plateau areas between the cavities on the surface. On these plateaus there are areas of slight irregularities as well as raised areas by overlapped an additional material. This may be seen in Fig. 6d. Usually the areas of higher irregularities tend to occur in the vicinity of cavities. Comparing the histograms Fig. 6c,g it can be noticed that the surface in the magnification micro zone is similar in term of surface flaws number. The occurrence of such flaws on the processed surface can be explained by the instability of plastic deformation processes occurring in the wave. The instability of the plastic deformation process in the jumping wave can be explained by the diversification of the material structure and inhomogeneity of the material properties, which may be seen in Fig. 7, where large clusters of grains of perlite and ferrite are visible. Figure 7 shows a cross-section through the burnished surface.

The outline of the sample surface creates pronounced serrations (2) of the structure of the material, which are the result of slippage of the material during the burnishing process. Figure 7b shows a magnification of the serrations seen in Fig. 7a. In this figure can be seen that the area between the serrations is filled by strongly deformed material. The deformation of this material is so large that the grains have been significantly fragmented and form alternating bands of metallographic structures existing in the processed material. The filling material of the serrated profile is consisted with significantly deformed structures and they were pressed with the surface of the sample. In Fig. 7 it is possible to see an expressed border between the core material behind these structures.

 Between neighboring structures there are also visible seams (item 6 in the Fig. 7). The structures marked 3 in Fig. 7 are generally connected to the surface of the sample by adhesion forces. The value of the adhesion forces is related to the degree of compression of this fragment with the surface. If these forces are small, such structures begin to separate from the surface, causing flaking. Photographs of a sample where flaking occur can be found in the literature^1,13^ which do not sufficiently explain the formation of such structures.

**Fig. 8:**
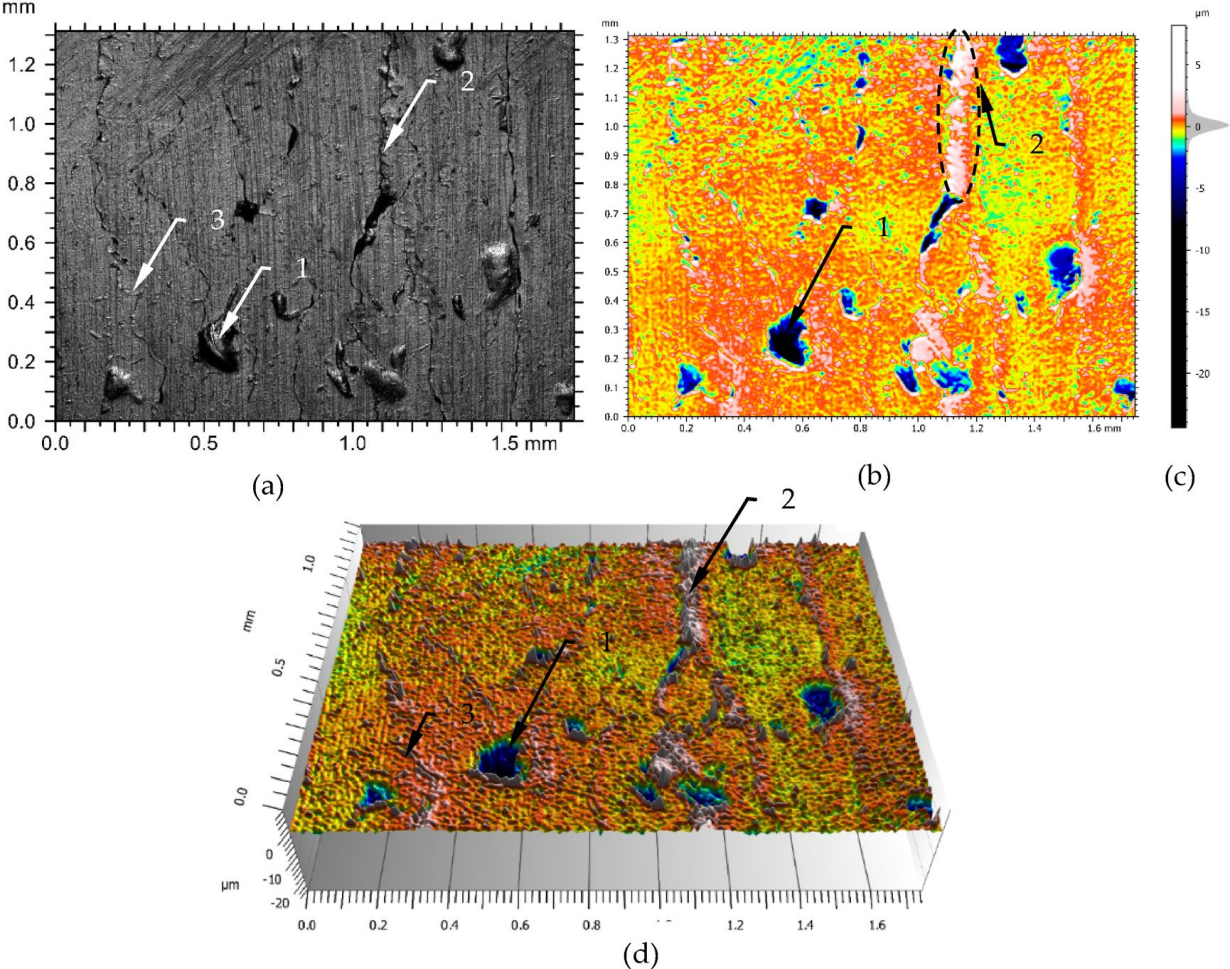
The surface structure after burnishing with a force of 800 N: (**a**) photograph of the surface, (**b**) profilograph, (**c**) scale and height distribution curve (**d**) 3D profilograph; 1- deep dale of the surface, 2- line of laps.

Figure 8 shows a surface that was burnished with a greater force than that of the machined sample in Fig. 4. The parameters used to carry out the process are listed in Table 3, line 2. Figure 8a shows the frontal photograph of the surface in which may be seen deep cavities (1), seams (2) and elements of strongly pressed material which resulted in the formation of serrated edges (3). The cavities shown in Fig. 8a generally lines up with the seam lines. The cavities are fewer in relation to Fig. 4 and the seams are more visible.

Comparing the amplitude density curve for Figs. 4 and 8, it can be seen that the spread of dominant values is smaller. Greater burnishing force resulted in this case in a greater degree of deformation of the material and on the surface, you can see more elements related to the high degree of cold rolling of the materials in the surface zone. Comparing the parameters in Tables 4 and 5 it can be seen that the values of Sa and Sq have decreased significant while the values of Sz, Sv, Sp have increased. It can be deduced that the increased pressing force causes slippage to a greater extent, which results in the formation of greater depth cavities and therefore increases the value of parameters such as Sv and Sz. Comparing Fig. 8a with Fig. 4a, it can be seen that the seams are less frequent on the surface shown in Fig. 8a than in Fig. 4a. On the other hand, elements of highly pressed material are present on the borders of these seams (Fig. 8a). The material is so highly pressed that a serrated edge is formed at the overlaps, which may be seen in item 2 in Fig. 8a, which is not present in Fig. 4a. It may be seen more similar structures on the surface if there are compared Figs. 4d and 8d.

**Table 5:** The 3D parameters of the surface burnished with a force 800 N, acc. ISO 25,178^31^.

Height parameters	Value	Unit	Description
Sq	1.21	µm	Root-mean-square height
Ssk	−4.76		Skewness
Sku	49.3		Kurtosis
Sp	8.19	µm	Maximum peak height
Sv	24.4	µm	Maximum dale height
Sz	32.6	µm	Maximum height
Sa	0.627	µm	Arithmetic mean height

**Fig. 9:**
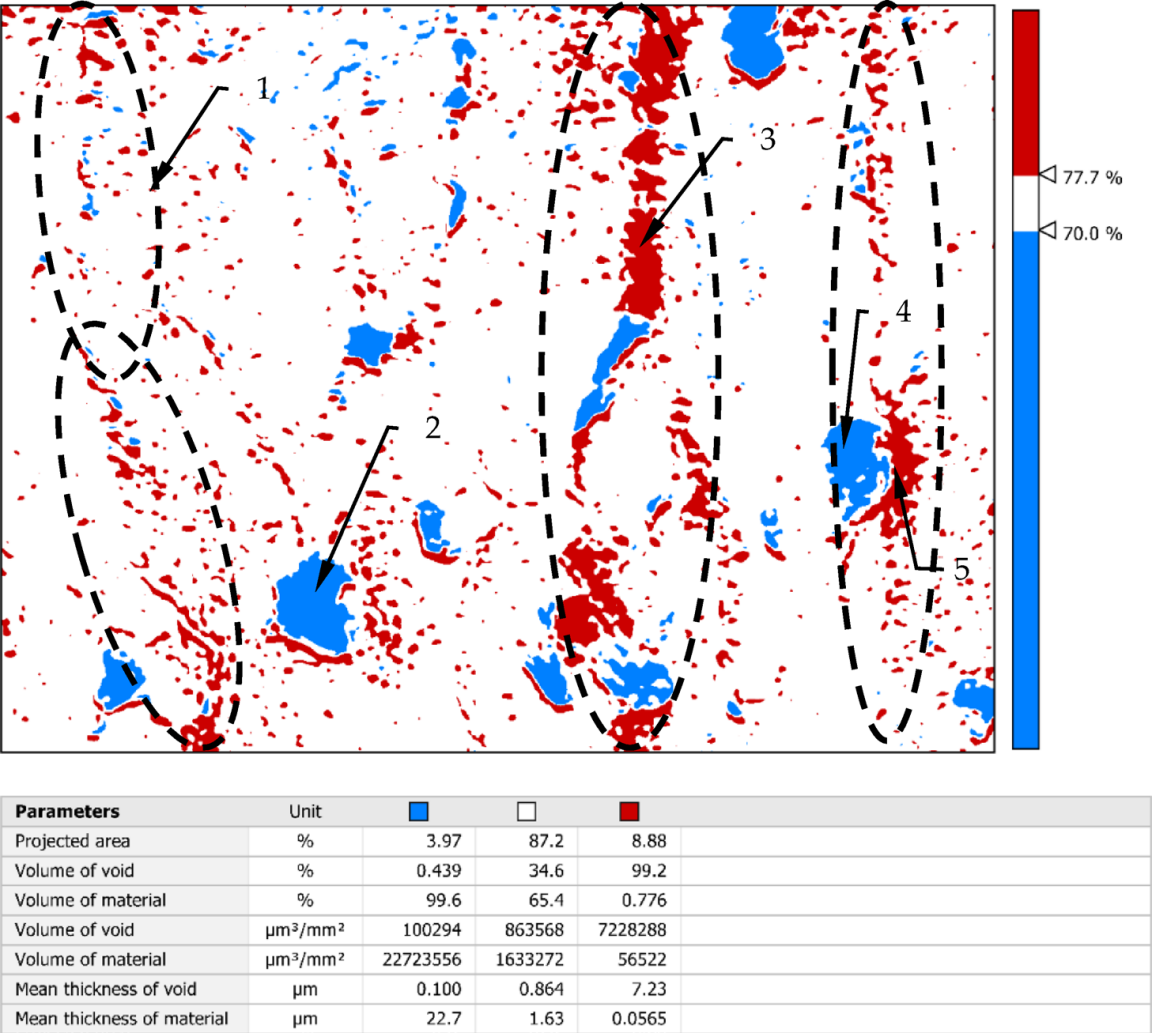
The peaks and dales structure the surface burnished with a force 800 N: 1- deep dale, 2- height peak, 3- areas of the layers slipping.

The analysis of peaks and cavities of the surface structure which was burnished with a force 800 N presents Fig. 9. In this analysis the mean value of the Sa parameter is about 0.63 μm. The thickness and position of the central zone relative to the range of highness roughness corresponds with the location of the amplitude density curve which is presented in the Fig. 8c. The analysis of peaks and cavities of the surface structure which was burnished with a force 800 N presents Fig. 9. In this analysis the mean value of the Sa parameter is about 0.63 μm. The thickness and position of the central zone relative to the range of highness roughness corresponds with the location of the amplitude density curve which is presented in the Fig. 8c. The central zone defined in this way occupies an area of 87.2% and is slightly larger than the corresponding zone in Fig. 5. The range of heights of irregularities included in this layer range from 70 to 77.7% of the height of the Sz parameter. Although the range of the depth of the cavities has been increased, they occupy a much smaller area compared to surface presented in Fig. 5. The cavities cover a smaller area, while with these machining parameters there are many small dales that were not occur in Fig. 5.

In Fig. 9a large number of peaks can be observed comparing to surface in the Fig. 5, but in this case must be taken into account the higher thickness of peaks zone. However, referring to the graph of the amplitude density curve in Fig. 8, the spread of the ordinates is smaller in relation to Fig. 4c, so, the higher number of peaks can be attributed mainly to the increasing of folds (flaws) on the surface. In Fig. 9 the dashed line marks the dominant peaks formation similarly to Fig. 5, it may be associated with the trace of the burnishing tool on the surface.

**Fig. 10:**
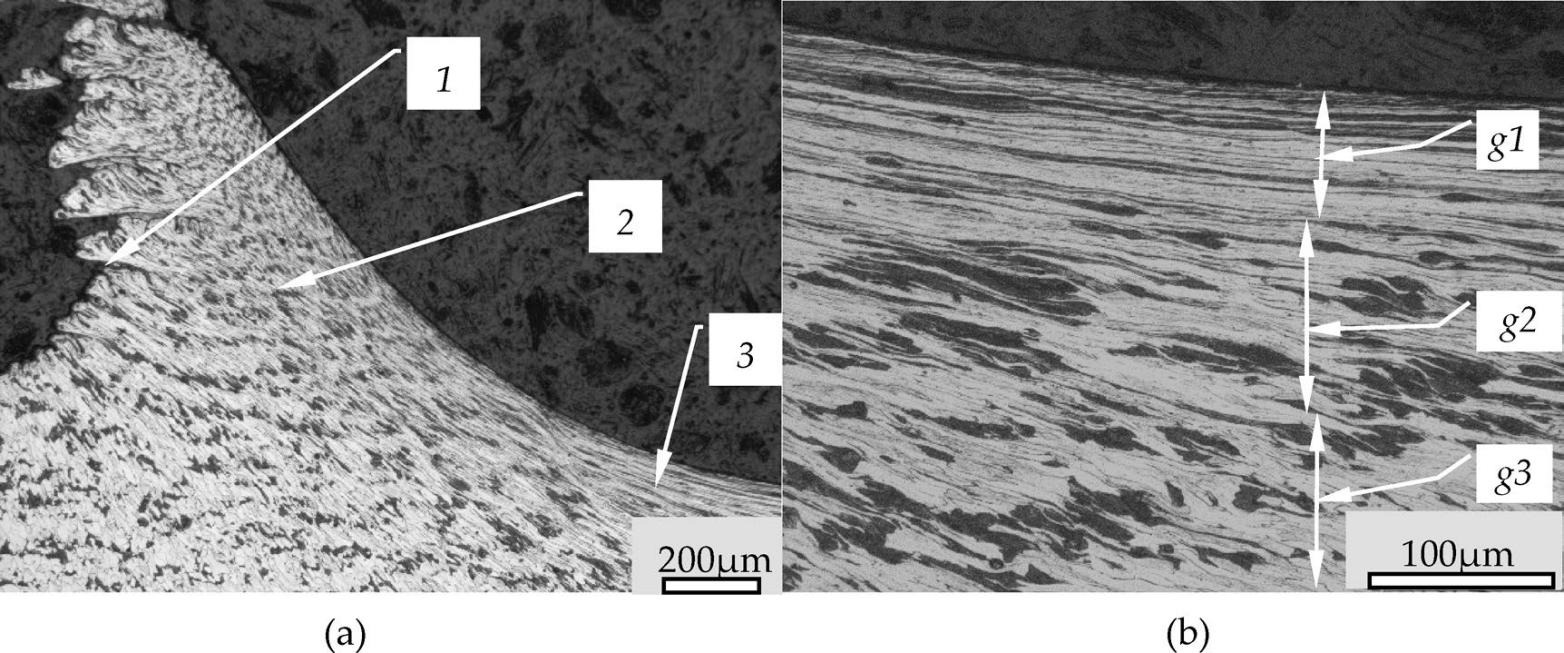
Jumping Wave section in front of the tool for a burnishing force of 1150 N: (**a**) wave view in front of the tool, (**b**) material structure under the tool; 1- material structures caused by slippage, 2- boundary of the post-sliding zone 3- deformation zone under the tool g1-g3 - the ranges of grain deformation in the under a tool zone.

**Fig. 11:**
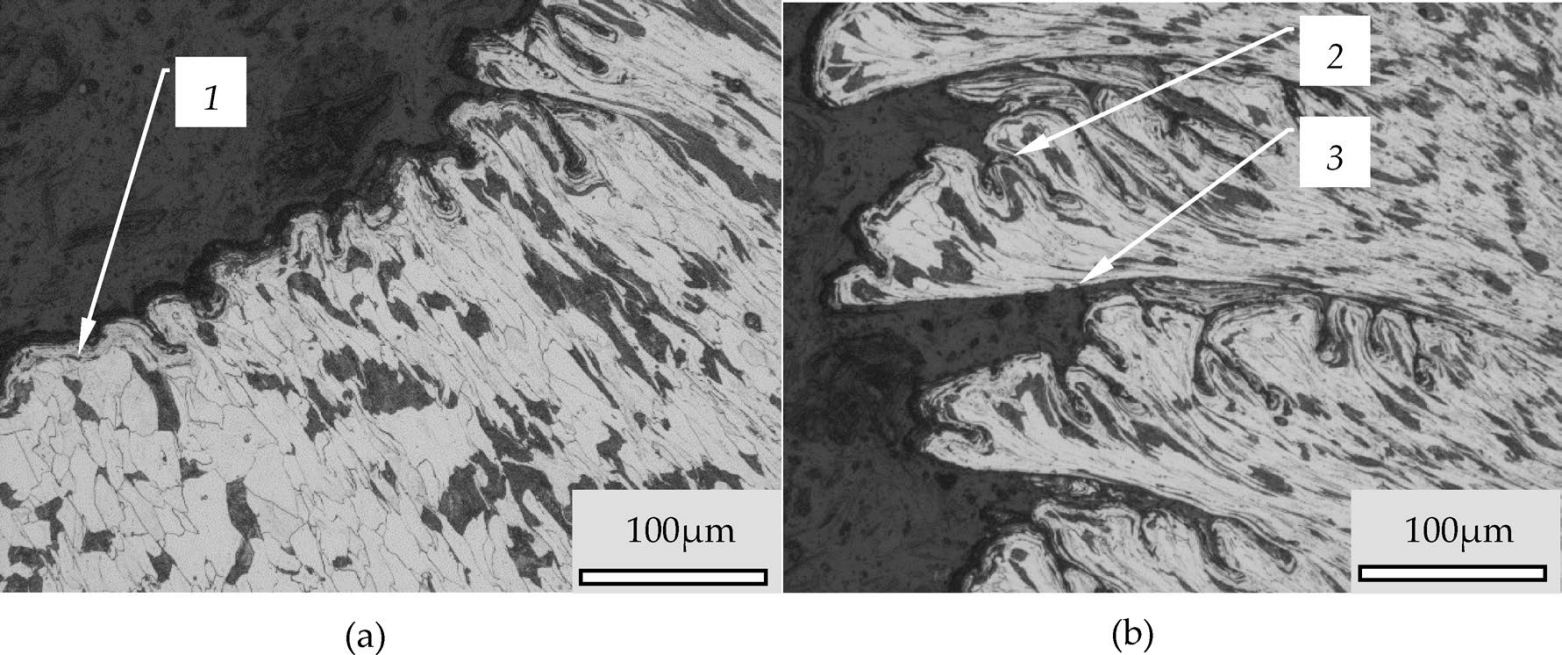
The forms of surface deformation occurring in the wave in front of the tool at an applied burnishing force of 1150 N: (**a**) the formation of irregularities in the slope of the wave, (**b**) high slippage of the material in the stuffing segment of the wave; 1- the initial phase of material displacement, 2, 3 - material structures at the slip planes.

Figure 10 shows a cross section of a wave that was created in front of the tool during the burnishing process after applying a force of 1150 N. This wave was not pulled under the burnishing tool but was pushing in front of the tool. This phenomenon caused the wave to increase which formed a ring around the roll. This ring was detached from the wave when it reached a certain height. In Fig. 10a, the line separating the ring from the wave may be seen in the upper left corner. The slipe effects of the material layers (item 1) in the wave may be seen in Fig. 8a, while on the tool side the surface is characterized by a high degree of smoothness. Observing the structure of the material one can notice orientation resulting from the slips, however, not causing deformation of the layers. On the other hand, in the wave from the side of tool interaction, deformations of material grains are visible, marked as 2 in Fig. 10a. In the tool bottom zone are occurred very large grain deformations (marked as 3 in Fig. 10a). The degree of grain deformation in this zone is shown in Fig. 10b. It may be seen that at different depths there is a variation in the amount of material grain deformation. The biggest deformation occurs in the (g1 zone) in Fig. 10b. Grains of ferrite and pearlite are deformed that form alternating narrow bands of specific structures. In (g2 zone) there is also significant deformation but the defragmentation of grains is much smaller. On the other hand, in (g3 zone) deformation of grains is insignificant but in this zone orientation of material structure is visible. Figure 11 presents the surface structure in the cross section through the wave of material in front of the tool. In this case the phenomenon was occurred after application pressing force of 1150 N. In this Figure, it may be observed the process of formation of additional irregularities on the surface due to movement of material layers in the wave. In Fig. 9a, the front surface of the wave is marked (1). In this Figure may be seen that the surface corrugation increases with the degree of deformation of the material in the wave. The degree of deformation may also be observed in the structure of the material. The left side of Fig. 9a shows a grain structure with a small degree of deformation. As the wave rises, the degree of deformation increases which may be seen in the shape of the grains of the material which are increasingly elongated. The orientation of the material structure is also noticeable. With the degree of deformation of the material structure, the number of irregularities on the surface increases, and it may be seen that mainly deep depressions begin to dominate. Figure 11b shows the structure of slipping in the pushing out material by the burnishing tool. In this case, such a slippage yields large changes in surface structure, while the way of movement of the material layers is interesting. In Fig. 11b one may notice a very unusual deformation resulting from large shearing forces. Heterogeneity of shear stresses causing slip create unusual deformations visible in the Fig. 11b. In the Figure may be seen that on the one side of the slip surface is significant corrugation and development of the slip surface (item 2), while the other side of the layer shows smooth (item 3) and this phenomenon is repeated in subsequent segments. The phenomena illustrated in Fig. 11 result in additional changes in the surface structure, which influence the machining results.

**Fig. 12:**
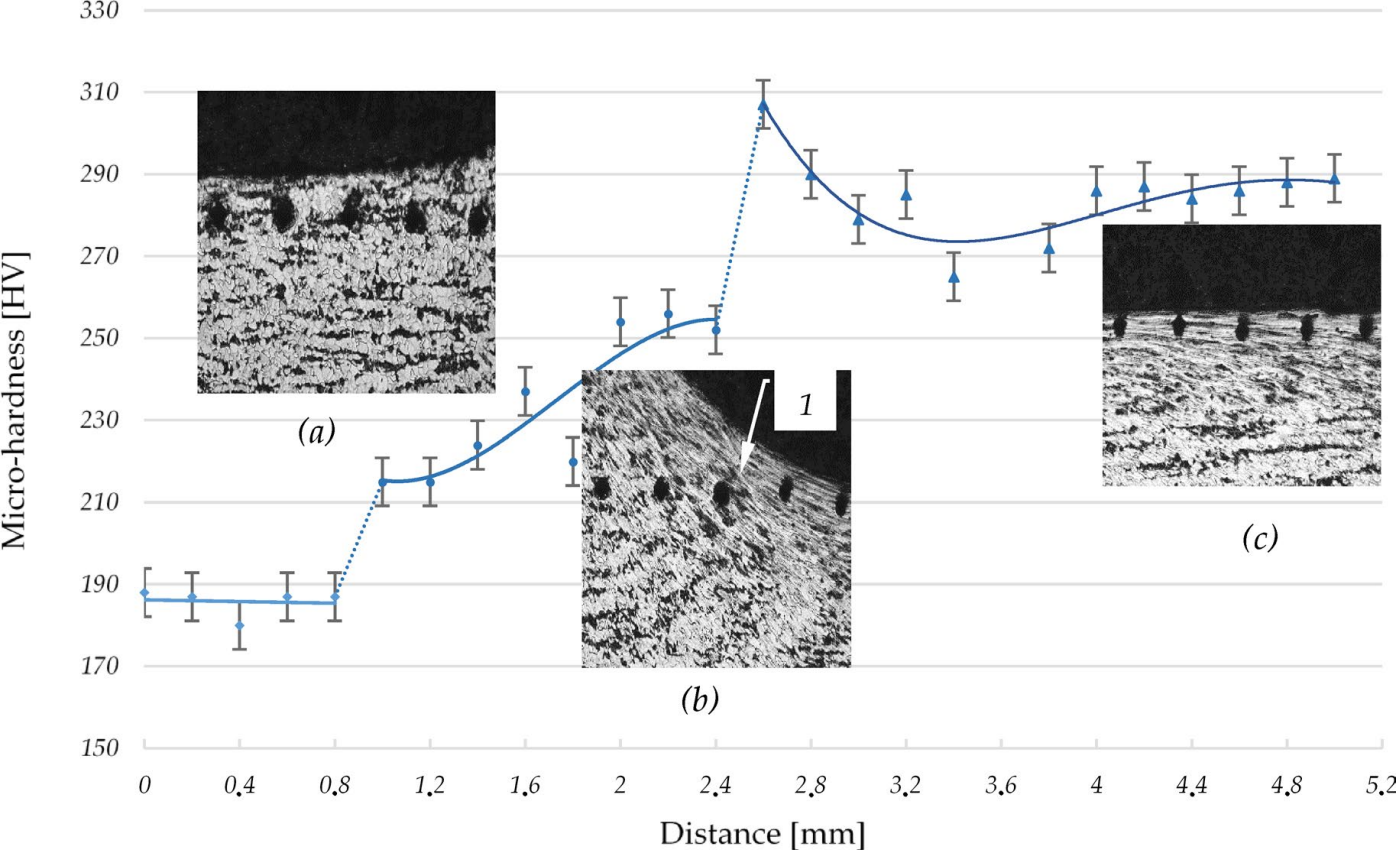
The distribution of surface micro-hardness occurring during burnishing process for force 1150 N: (**a**) micro-hardness tool indentations before jumping wave, (**b**) micro-hardness tool indentations in the wave and under the burnishing tool, (**c**) micro-hardness tool indentations after burnishing processing; 1- material structure line.

A micro-hardness measurement was performed for the wave shown in Fig. 12. The measurement was realized on the distance of 5 mm in the area including the wave and its surroundings. Individual measurements were performed every 0.2 mm.

The device on which the measurements were performed was initially checked in terms of stability of the obtained results and on this basis the standard deviation for the measured values was calculated and plotted on Fig. 10 for each measurement point. In the Fig. 12 in the first section 0 ÷ 0.8 mm the surface had a structure after turning, and the micro-hardness results were about 188HV (Vickers grade).

The surface is slightly harder in relation to the core (its hardness was in the range 130 ÷ 150HV depending on the proportion of ferrite and pearlite grains occurring in a given place). Hardness increase observed is due to the turning process as a result of which slightly changes occurred in the surface layer of the element. Between points 0.8 ÷ 1 mm, material deformation occurs in the jumping wave moving before burnishing tool. The shear stresses causing deformation of the workpiece result in built-up in the processing material: dislocations, twins, layers slip, etc. which results in an increase in material hardness in the wave.

Figure 12b shows traces of hardness measurements at these points. The fragment of the graph between points 1 and 2.4 mm shows the increase of hardness in relation to the intensity of material deformation in the wave. The deformation process is unstable and local area can form in the material which show low hardness. High burnishing force (1150 N) applied in this case caused significant deformation of the material grains under the tool. This deformation causes a significant increase in hardness which can be seen between the values of 2.4 mm and 2.6 mm in Fig. 12. In the photograph (b) between these points a line (item 1) of significant deformation of material grains is visible. The influence of the tool pressing, there are very high pressures in this area respect causing the burnished material to flow. This phenomenon introduces changes in the structure of material hardness, which can be observed on the graph between 2.6 mm and 4 mm. The points in the 4 ÷ 5 mm range show stability in micro-hardness, this is basically the surface after burnishing. Traces of hardness measurements in this zone are shown in Fig. 12c.

## Conclusions

During low plastic burnishing process of the soft materials usually occur jumping wave in front of the processing tool. This wave causes deformations of the burnished surface mainly in the form of slippage between the internal layers of the material, which creates additional irregularities on the processing surface of the workpiece. Increasing the burnishing force increases the value of the material cold rolling which generally reduces the value of surface irregularities highness, on the other hand, the number of surface flaws in the form of folds and deep of cavities on the surface are increased.

The significant results of the experimental research can be summarized as follows:

- In the research, deformed folds were observed on the workpiece surface, with serrated edges clearly visible, and their raised portions creating additional surface structures (defects). These folds are pressed into the surface, connecting it mainly through adhesive forces. Higher burnishing force causes increased slippage of the surface layers, which results in the destruction of the remaining bonds. At the same time, the adhesive forces holding the structure together are too weak, so fragments of material easily detach from the surface, leading to flaking phenomena.
- Increasing the pressing force from 550 N to 800 N significantly reduced the area of irregularities remaining on the surface from 15.8 to 3.98%. On the other hand, it was observed that in the case of burnishing with a higher force, although the Sa parameter was significantly reduced from 1.89 μm to 0.63 μm, the remaining cavities have a much greater depth. The Sz parameter increased from 21.1 μm to 32.6 μm, while the Sv parameter increased from 14.5 μm to 24.4 μm, and the Sp parameter increased from 6.46 μm to 8.18 μm.
- Characterizing the surface after burnishing with a skewness parameter index Ssk the change occurs from − 1.86 (for force 550 N) to 4.76 (for force 800 N) which means that the amplitude density curve becomes dominated by cavities. While kurtosis index Sku increases from 7.47 to 49.3 which means that the amplitude density curve became narrows considerably and the probability of occurrence of extreme values of profile ordinates decreased significantly.

Further research work will be focused on the investigation in to jumping wave occurring phenomena in the burnishing process realized under differentiated machining conditions.

## Data Availability

All data generated or analysed during this study are included in this published article.
